# TGFβ and EGF signaling orchestrates the AP-1- and p63 transcriptional regulation of breast cancer invasiveness

**DOI:** 10.1038/s41388-020-1299-z

**Published:** 2020-04-29

**Authors:** Anders Sundqvist, Eleftheria Vasilaki, Oleksandr Voytyuk, Yu Bai, Masato Morikawa, Aristidis Moustakas, Kohei Miyazono, Carl-Henrik Heldin, Peter ten Dijke, Hans van Dam

**Affiliations:** 1 Department of Medical Biochemistry and Microbiology, Science for Life Laboratory, Uppsala University, SE 751 23 Uppsala, Sweden; 2 Department of Molecular Pathology, Graduate School of Medicine, The University of Tokyo, Tokyo, 113-0033 Japan; 3 Department of Cell and Chemical Biology, Oncode Institute, Leiden University Medical Center, 2300 RC Leiden, The Netherlands

**Keywords:** Breast cancer, Breast cancer, Growth factor signalling, Growth factor signalling

## Abstract

Activator protein (AP)-1 transcription factors are essential elements of the pro-oncogenic functions of transforming growth factor-β (TGFβ)-SMAD signaling. Here we show that in multiple HER2+ and/or EGFR+ breast cancer cell lines these AP-1-dependent tumorigenic properties of TGFβ critically rely on epidermal growth factor receptor (EGFR) activation and expression of the ΔN isoform of transcriptional regulator p63. EGFR and ΔNp63 enabled and/or potentiated the activation of a subset of TGFβ-inducible invasion/migration-associated genes, e.g., *ITGA2, LAMB3*, and *WNT7A/B*, and enhanced the recruitment of SMAD2/3 to these genes. The TGFβ- and EGF-induced binding of SMAD2/3 and JUNB to these gene loci was accompanied by p63-SMAD2/3 and p63-JUNB complex formation. p63 and EGFR were also found to strongly potentiate TGFβ induction of AP-1 proteins and, in particular, FOS family members. Ectopic overexpression of FOS could counteract the decrease in TGFβ-induced gene activation after p63 depletion. p63 is also involved in the transcriptional regulation of heparin binding (HB)-EGF and EGFR genes, thereby establishing a self-amplification loop that facilitates and empowers the pro-invasive functions of TGFβ. These cooperative pro-oncogenic functions of EGFR, AP-1, p63, and TGFβ were efficiently inhibited by clinically relevant chemical inhibitors. Our findings may, therefore, be of importance for therapy of patients with breast cancers with an activated EGFR-RAS-RAF pathway.

## Introduction

Transforming growth factor-β (TGFβ)-induced signaling has both positive and negative functions in cancer; in late phases TGFβ frequently stimulates tumor cell invasion and metastasis. Most of these functions are mediated through SMADs, which are the canonical intracellular transcriptional effectors of the TGFβ receptors. Activated SMADs form complexes with other DNA binding transcription factors to elicit cell-type-dependent responses. Cancers are characterized by tumor- and patient-specific de-regulation of multiple signaling pathways, and, in addition, generation of specific and altered interactions with the microenvironment relative to the normal tissue organization. Understanding the complex interplay of oncogenic signaling pathways, including antagonistic, cooperative, and synergistic effects, is critical to understand the nature of cancer cell phenotypes, and to enable more efficient and specific therapeutic intervention. The combined action of epidermal growth factor (EGF) and TGFβ signaling represents a classic example of oncogenic cooperation and context-dependence. TGFβ was initially discovered by its ability to induce normal rat kidney (NRK) cells to grow in soft agar in cooperation with TGFα or EGF [1, 2]. In normal epithelial cells EGF induces proliferation, whereas TGFβ acts as a growth inhibitor, by inducing cell cycle arrest and apoptosis. However, both EGF and TGFβ can trigger epithelial-mesenchymal transition (EMT) and migratory responses in epithelial cells [3–7]. TGFβ can become a strong tumor promoter in cancer cells that have become insensitive to TGFβ-induced growth inhibition by MYC activation and other cell cycle defects. In particular this is the case in the presence of additional pro-oncogenic signals, such as high levels of active EGFR, mutant RAS and WNT-β-CATENIN signaling [4–7]. In specific circumstances, this may involve direct counteraction of TGFβ’s tumor suppressive effects by HER2 overexpression [8, 9].

Signaling by TGFβ occurs via type I and type II serine/threonine kinase receptors (TGFβRI and TGFβRII, respectively), which mainly propagate the signal through phosphorylation of the receptor-regulated (R-) SMAD proteins, i.e., SMAD2 and SMAD3 [10, 11]. Activated R-SMADs form complexes with common-partner (Co-) SMAD, i.e., SMAD4. These heteromeric complexes accumulate in the nucleus and control gene expression in a cell-type- and gene-specific manner. Essential for the specificity are the interactions of SMADs with lineage-determining and signal-driven transcription factors, chromatin-remodeling factors, co-activators, and co-repressors, which increase SMAD DNA binding and transactivating potential. In addition to these interactions, multiple other layers of regulation influence the intensity and duration of TGFβ signaling, and thereby define the specificity of the response [7, 12]. This fine-tuning involves amongst others various ubiquitin E3 ligases and non-SMAD signaling pathways, such as the PI3K-AKT-mTOR and MAPK pathways, which can be induced by TGFβ as well as other growth-regulatory stimuli [4, 12].

EGF receptor activation can potently trigger multiple kinase cascades, including the RAS-MAPK and the PI3K-AKT pathways, and thereby enhance survival, proliferation, differentiation, and motility [3]. Cross-talk between EGFR and TGFβ signaling occurs at multiple levels, for instance via post-translational modification of the SMAD proteins [4, 5, 13, 14], and by induction of each other´s ligands (HB-EGF, TGFβ1), which results in sustained activation of the MAPK and PI3K-AKT pathways [9]. However, the effects of this cross-talk on the interplay between SMAD proteins and SMAD-cooperating transcription factors in tumor-promotion are relatively unknown.

The members of the AP-1 family of transcription factors regulate gene expression in response to a large number of stimuli and pathways, including TGFβ-SMAD and EGFR-MEK-MAP kinase signaling. The “classical” AP-1 family is composed of dimers of JUN, JUNB or JUND, and FOS, FOSB, FOSL1 or FOSL2, which all exhibit specific functions in the control of cell proliferation and differentiation [15–22]. Certain AP-1 components have been implicated in tumor cell invasion [23–25], and in particular FOSL1 has been associated with breast cancer metastasis, EMT and cancer stemness [26–29]. Various JUN and FOS members have been found to interact with SMADs [25, 30, 31], and JUNB triggers activation of a TGFβ-induced SMAD-dependent breast cancer invasion program [25, 32].

The p53 family member p63 is a transcriptional regulator of epithelial development and differentiation, acting as a common downstream effector of activated EGFR/RAS and TGFβ pathways, and playing an important role in primary breast cancers. p63 can be expressed in multiple different protein isoforms (TAp63α/β/γ/δ/ε and ΔNp63α/β/γ/δ/ε), of which the ΔN forms lack an intact transactivation (TA) domain. TAp63 and ΔNp63 isoforms can have dual, gene-specific but opposite, effects on target genes [33], implying that their expression needs to be finely regulated during cancer initiation and progression. ΔNp63 isoform is frequently overexpressed in human malignancies and can regulate oncogenic routes involved in the pathogenesis of breast carcinoma by contributing to proliferation, stemness and survival of breast tumors [34]. Previously, we demonstrated, on a genome-wide scale, that co-activation of RAS and TGFβ signaling via downregulation of mutant p53 can enhance binding of p63 to its genomic sites [35]. In this paper, we present novel mechanistic insight into the pro-oncogenic EGF-TGFβ-p63-AP-1 interplay in breast cancer cells.

## Results

### TGFβ requires co-stimulation of EGFR to induce a pro-invasive gene program in HER2+ and EGFR+ breast cancer cells

**Fig. 1:**
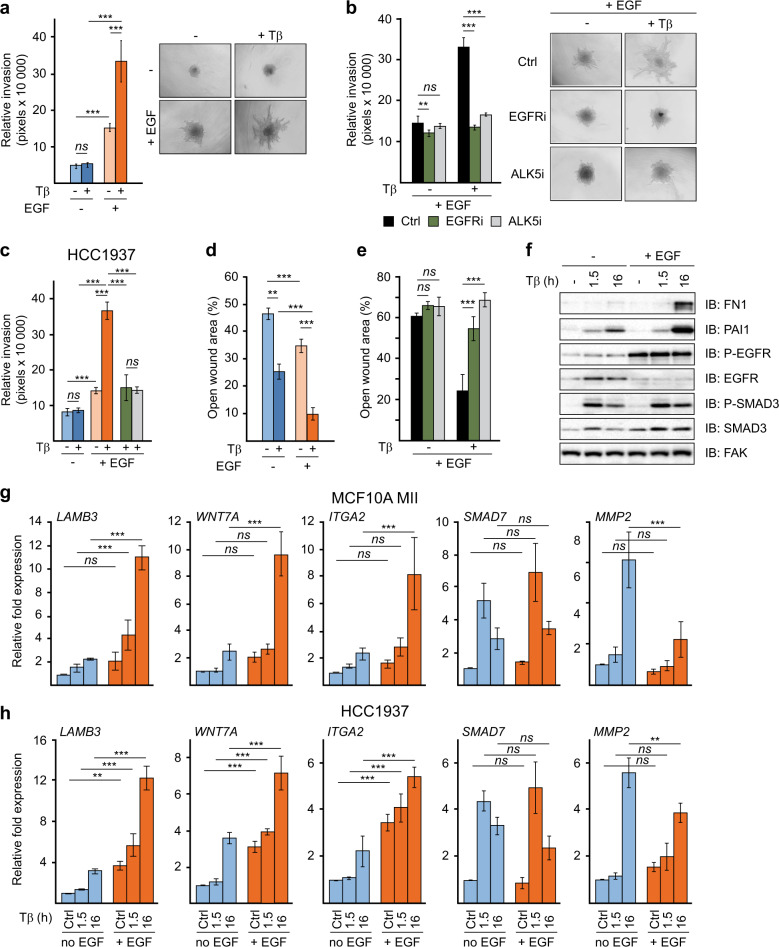
TGFβ-induced invasion and migration requires EGFR activity. **a** Collagen-invasion of MCF10A MII spheroids in the presence or absence of 5 ng/ml TGFβ1 (Tβ) and 20 ng/ml EGF, as indicated. Left: relative invasion was quantified as the mean area that the spheroids occupied 28 h after being embedded in collagen. Right: representative pictures of spheroids were taken 28 h after embedding. **b** The effects of the EGFR inhibitor lapatinib (EGFRi; 10 µM) and the TGFβRI inhibitor SB505124 (ALK5i; 2.5 µM) on 5 ng/ml TGFβ1-induced collagen-invasion of MCF10A MII spheroids in the presence of 20 ng/ml EGF. Left: relative invasion was quantified as the mean area that the spheroids occupied 28 h after being embedded in collagen. Right: representative pictures of spheroids were taken 28 h after embedding. **c** Collagen-invasion of HCC1937 spheroids in the presence or absence of TGFβ1 (5 ng/ml), EGF (20 ng/ml), lapatinib (EGFRi) and SB505124 (ALK5i) as indicated, performed as described under (**a**, **b**). **a**–**c** Statistics were calculated using one-way analysis of variance (ANOVA). The data were further analyzed using Tukey’s multiple comparisons test. Data represent mean ± SD (*n* ≥ 6 spheroids per condition) and are representative of three independent experiments; *ns*, not significant, **P < 0.01, and ***P < 0.001. **d** Migration of MCF10A MII cells in the presence or absence of TGFβ and EGF measured by wound-healing (scratch) assays. MCF10A MII cells were incubated for 16 h in 0.2% FBS starvation medium in the presence or absence of 20 ng/ml EGF, and then treated with 5 ng/ml TGFβ1 as indicated. Migration was measured after 48 h by quantifying the percentage of open area left after scratching a confluent cell layer at *t* = 0. **e** The effects of the EGFR inhibitor lapatinib (1 µM) and the TGFβRI inhibitor SB505124 (2.5 µM) on TGFβ + EGF-induced migration of MCF10A MII cells measured by wound-healing (scratch) assays. Cells were incubated for 16 h in 0.2% FBS starvation medium in the presence of 20 ng/ml EGF before addition of 5 ng/ml TGFβ1 and/or inhibitors, as indicated. Migration was measured after 42 h by quantifying the percentage of open area left after scratching a confluent cell layer at *t* = 0. **d**–**e** Statistics were calculated using one-way analysis of variance (ANOVA). The data were further analyzed using Tukey’s multiple comparisons test. Data represent mean ± SD (*n* = 8 measurements per sample) and are representative of three independent experiments; *ns*, not significant, **P < 0.01, and ***P < 0.001. **f** Western blot analysis of TGFβ–EGF cooperation. MCF10A MII cells were incubated for 16 h in starvation medium containing EGF, insulin, cholera toxin, hydrocortisone, 0.2% FBS, or in starvation medium lacking EGF (20 ng/ml), and subsequently treated with 5 ng/ml TGFβ1 for 0, 1.5, or 16 h, as indicated and analyzed by immunoblotting. One of three independent experiments with similar results, is shown. **g**, **h** EGF enables and enhances activation of a subset of TGFβ migration/invasion genes. qRT-PCR analysis of TGFβ- and EGF-induced gene expression. MCF10A MII (**g**) or HCC1937 (**h**) cells were treated as in **f**. Statistics were calculated using one-way analysis of variance (ANOVA). The data were further analyzed using Tukey’s multiple comparisons test. Results from four independent experiments are shown as mean ± SD; *ns*, not significant, **P < 0.01, and ***P < 0.001.

Using a 3D model of collagen-embedded spheroids of premalignant MCF10A human breast cancer cells, we previously found TGFβ-SMAD signaling to induce breast cancer cell invasion by activating various invasion-associated genes, including matrix metallo-proteinases (MMPs) and WNT family members [25, 32, 36]. The T24 H-RAS-transformed MCF10A MII cells used in these studies are routinely cultured in the presence of EGF, insulin and other supplements, since parental MCF10A cells require growth factors and hormones to proliferate [37, 38]. Since both active RAS and EGFR signaling can influence TGFβ-induced functions, we determined the pro-invasive effect of TGFβ in the 3D spheroid system in absence and presence of EGF. Surprisingly, TGFβ induced invasion only in the presence of EGF, whereas EGF had a weak invasive effect by itself (Fig. 1a). Moreover, TGFβ-induced invasion in the presence of EGF was inhibited both by the EGFR kinase inhibitor lapatinib and by the TGFβRI kinase (ALK5) inhibitor SB505124 (Fig. 1b). Similar results were obtained for the basal EGFR+ breast cancer cell line HCC1937 (Fig. 1c and Supplementary Fig. S1a). When analyzed for their migratory properties in 2D wound-healing assays, MCF10A MII cells were found to migrate most efficiently when both TGFβ and EGF were present (Fig. 1d and Supplementary Fig. S1b). The TGFβ-induced migration in the presence of EGF was under these conditions also inhibited by either inhibition of the EGFR kinase or the TGFβRI kinase (Fig. 1e and Supplementary Fig. S1c). Analysis of some of the major TGFβ-inducible proteins also showed dependence on EGF. Fibronectin 1 (FN1) was only induced by TGFβ in the presence of EGF in MII cells, and the late TGFβ induction of plasminogen activator inhibitor 1 (PAI1, encoded by the gene *SERPINE1*) was enhanced under these conditions. The induction of C-terminal SMAD3 phosphorylation by TGFβ was not appreciably affected by EGF, and autophosphorylation of EGFR-Tyr1063 that was strongly enhanced by EGF, was not affected by TGFβ (Fig. 1f).

To investigate the mechanism by which EGFR signaling enables and potentiates TGFβ-induced invasion and migration, we analyzed the effect of EGF on the expression of previously identified TGFβ-inducible invasion genes [25, 32, 36]. In MII cells a subset of them, including *LAMB3, ITGA2, WNT7A*, *WNT7B*, and *MMP10*, were induced by TGFβ only when EGF was present, whereas others, such as *MMP2* and *SNAI1*, were induced by TGFβ in the absence of EGF; other genes were hardly affected, such as *COL7A1* and *SMAD7* (Fig. 1g and Supplementary Fig. S1d). In the EGFR+ HCC1937 cells, and also in basal-like EGFR+ and HER2+ HCC1954 breast cancer cells, the presence of EGF strongly enhanced both the basal and TGFβ-induced levels of subsets of these invasion genes, whereas HER2+ HCC202 cells showed similar result as MII cells (Fig. 1h and Supplementary Fig. S1e, f).

We next examined the effect of EGF on the binding of SMAD2/3 to representative SMAD binding regions, which we had previously identified by chromatin immunoprecipitation (ChIP) sequencing [32]. ChIP analysis in MCF10A MII cells showed that the TGFβ-induced binding of SMAD2/3 to the *LAMB3*, *WNT7B*, and *ITGA2* SMAD binding regions was strongly enhanced by EGF, whereas SMAD2/3 binding to the *SMAD7* region was not appreciably affected, and the binding to *MMP*2 was reduced (Supplementary Fig. S1g). These SMAD2/3 ChIP results thus correlate with the TGFβ-induced mRNA levels of the respective genes in the presence and absence of EGF, and show that the effects of EGF on TGFβ-dependent invasion/migration genes are gene locus specific, and therefore may depend on the presence of specific transcription factors cooperating with SMADs. In summary, these result show that in multiple HER2+ and/or EGFR+ breast cancer cell lines EGF selectively enables and/or potentiates activation of a subset of critical TGFβ-SMAD inducible invasion/migration-associated genes.

### EGFR can cooperate with TGFβRI via the MEK–ERK pathway

**Fig. 2:**
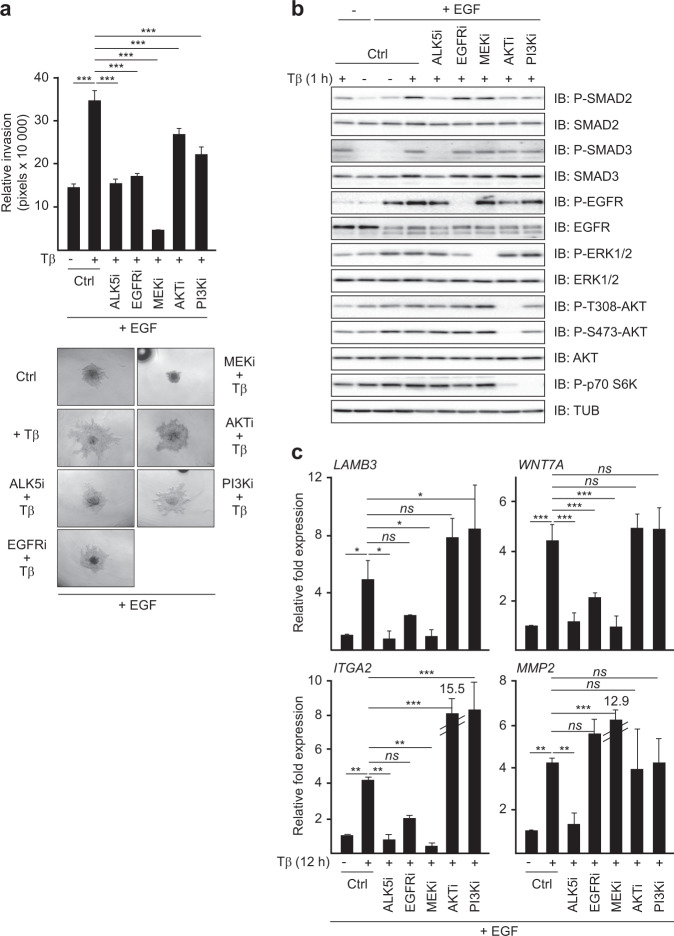
EGFR cooperates with TGFβRI via the MEK–ERK pathway. **a** Comparison of the kinase inhibitors SB505124 (ALK5i), lapatinib (EGFRi), AZD6244 (MEKi), MK-2206 (AKTi), and LY294002 (PI3Ki) on TGFβ1-induced collagen-invasion of MCF10A MII spheroids in the presence of EGF (20 ng/ml). Top: relative invasion was quantified as the mean area that the spheroids occupied 26 h after being embedded in collagen. Statistics were calculated using one-way analysis of variance (ANOVA). The data were further analyzed using Dunnett’s multiple comparisons test and compared with the results from cells treated with TGFβ1 (5 ng/ml) alone. Data represent mean ± SD (*n* ≥ 6 spheroids per condition) and are representative of three independent experiments; *ns*, not significant, ***P < 0.001. Bottom: representative pictures of spheroids were taken 26 h after embedding. **b** Immunoblot validation of kinase inhibitor specificity. MCF10A MII cells were incubated for 16 h in 0.2% FBS starvation medium with or without EGF (20 ng/ml) before addition of the same kinase inhibitors as in (**a**), or control. 15 min later TGFβ1 (5 ng/ml) was added and incubation prolonged for 1 h. One of three independent experiments with similar results, is shown. **c** qRT-PCR analysis of TGFβ and EGF-induced gene expression. MCF10A MII cells were incubated for 16 h in 0.2% FBS starvation medium with EGF (20 ng/ml) before addition of the indicated kinase inhibitors or DMSO (control). TGFβ1 (5 ng/ml) was added 15 min later and incubation prolonged for 12 h. Statistics were calculated using one-way analysis of variance (ANOVA). The data were further analyzed using Dunnett’s multiple comparisons test and compared with the results from cells treated with TGFβ1 (5 ng/ml) alone. Results from three independent experiments are shown as mean ± SD; *ns*, not significant, *P < 0.05, **P < 0.01, and ***P < 0.001.

EGFR activates multiple downstream signaling pathways, including the RAF-MEK-ERK and the PI3K-AKT pathways, both of which cooperate with TGFβ-SMAD-signaling and gene activation [9, 14]. To examine the role of these pathways in TGFβ-induced MII spheroid invasion in collagen in the presence of EGF, we used two different MEK inhibitors PD184352/CL**-**1040 and AZD6244 (Selumetinib), the PI3K inhibitor LY294002, and the AKT inhibitor MK-2206 and compared their effects with those of the EGFR and TGFβRI kinase inhibitors. Similar as in Fig. 1a, the TGFβRI and EGFR kinase inhibitors completely counteracted TGFβ + EGF-induced invasion, whereas the PI3K and AKT inhibitors only had weak suppressive effects. Inhibition of the MEK-ERK pathway showed much stronger effects than TGFβ or EGFR kinase inhibition and completely blocked both basal and TGFβ-induced invasion of MCF10A MII spheroids embedded in collagen in the presence of EGF (Fig. 2a and Supplementary Fig. S2a, b).

To validate these differential effects and verify the specificity of the inhibitors, we next analyzed the activity state of downstream targets of the involved pathways. As shown in Fig. 2b and Supplementary Fig. S2c, the TGFβRI kinase inhibitors SB505124 and LY364947 strongly inhibited the C-terminal phosphorylation of SMAD2 and SMAD3 induced after 1 h of TGFβ-treatment, but not the levels of active, phosphorylated EGFR, ERK, AKT, and p70 S6 kinase. Conversely, lapatinib strongly reduced the auto-phosphorylation of EGFR, and partially reduced phospho-ERK and the phospho-p70 S6K control, but did not affect C-terminally phosphorylated SMAD2 and SMAD3. In contrast, the MEK inhibitors PD184352 and AZD6244 completely inhibited the phosphorylation of the MEK substrate ERK (Fig. 2b and Supplementary Fig. S2c), but had no effects on the other targets. As expected, the AKT inhibitor MK-2206 completely reduced the (auto) phosphorylation of AKT and partially reduced the phosphorylation of p70 S6 kinase (Fig. 2b). The PI3K inhibitor LY294002 completely blocked the phosphorylation of p70 S6K and partially reduced the phosphorylation of AKT, but similar to the AKT inhibitor, did not affect ERK phosphorylation (Fig. 2b and Supplementary Fig. S2c). These results thus confirm the efficiency and pathway specificity of these inhibitors at the conditions used.

Interestingly, the two MEK inhibitors reduced the levels of phospho-ERK much stronger than the EGFR inhibitor, while the PI3K and AKT inhibitors had more potent effects on phospho-AKT and p70 S6K than EGFR inhibition (Fig. 2b and Supplementary Fig. S2c). These observations suggest that EGFR is not the only upstream activator of MEK-ERK and PI3K-AKT in the mutant RAS expressing MII cells, and may also explain the very strong inhibitory effects of the MEK inhibitors on both basal and TGFβ-induced invasion of MCF10A MII spheroids in EGF containing medium.

We next compared the effects of TGFβRI, EGFR, MEK-ERK and PI3K/AKT inhibition on the EMT- and invasion-associated genes that in Fig. 1g showed different TGFβ-SMAD responses in the presence and absence of EGF. The TGFβRI, EGFR and MEK inhibitors, but not the PI3K or AKT inhibitors, strongly reduced the mRNA levels of *LAMB3*, *WNT7A*, and *ITGA2* (Fig. 2c). In contrast, the induction of *MMP2*, which was reduced by EGF (Fig. 1g), was inhibited by the TGFβRI inhibitor, but enhanced rather than suppressed by the EGFR and MEK inhibitors (Fig. 2c). Moreover, similar to ERK phosphorylation and TGFβ-induced collagen-invasion, the induction of *LAMB3, WNT7A* and *ITGA2* by TGFβ in the presence of EGF was more strongly inhibited by MEK inhibition than by EGFR inhibition. Together, these results indicate that the EGFR-MEK-ERK arm can play a critical role in TGFβ-induced invasion by enabling and/or strongly potentiating TGFβ-induction of selective invasion/migration-associated genes.

### EGFR signaling enables and potentiates TGFβ induction of AP-1 (JUN/FOS)

**Fig. 3:**
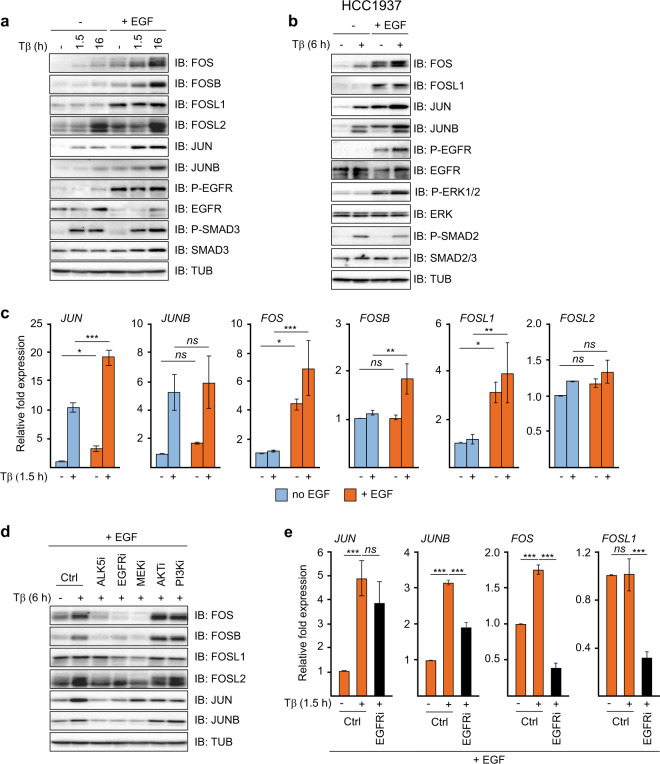
EGFR-MEK signaling enables TGFβ induction of FOS family components. **a** Immunoblot analysis of TGFβ- and EGF-induced AP-1 components. MCF10A MII cells were incubated for 16 h in 0.2% FBS starvation medium with or without EGF (20 ng/ml) and subsequently treated with 5 ng/ml TGFβ1 for 0, 1.5, or 16 h, as indicated. One of three independent experiments with similar results, is shown. **b** Immunoblot analysis of TGFβ- and EGF-induced AP-1 components. HCC1937 cells were incubated for 16 h in 0.2% FBS starvation medium with or without EGF (20 ng/ml) and subsequently treated with 5 ng/ml TGFβ1 for 6 h as indicated. One of three independent experiments with similar results, is shown. (**c**) qRT-PCR analysis of TGFβ- and EGF-induced mRNA for JUN and FOS family members. MCF10A MII cells were treated as in (**a**). Statistics were calculated using one-way analysis of variance (ANOVA). The data were further analyzed using Tukey’s multiple comparisons test. Results from three independent experiments are shown as mean ± SD; *ns*, not significant, *P < 0.05, **P < 0.01, and ***P < 0.001. **d** The effects of the kinase inhibitors 2.0 µM LY364947 (ALK5i), lapatinib (EGFRi), AZD6244 (MEKi), MK-2206 (AKTi), and LY294002 (PI3Ki) on TGFβ- and EGF-induced AP-1 components. MCF10A MII cells were incubated for 16 h in 0.2% FBS starvation medium with EGF (20 ng/ml), before addition of the indicated kinase inhibitors or DMSO (control). 15 min later TGFβ1 (5 ng/ml) was added and incubation prolonged for 6 h and cells were then analyzed by immunoblotting. One of three independent experiments with similar results, is shown. **e** qRT-PCR analysis of the effect of EGFR inhibition on TGFβ- and EGF-induced *JUN* and *FOS* mRNAs. MCF10A MII cells were incubated for 16 h in 0.2% FBS starvation medium with EGF (20 ng/ml) before addition of lapatinib (EGFRi) or DMSO (control). 15 min later TGFβ1 (5 ng/ml) was added and incubation prolonged for 1.5 h. Statistics were calculated using one-way analysis of variance (ANOVA). The data were further analyzed using Dunnett’s multiple comparisons test and compared with the results from cells treated with TGFβ1 alone. Results from four independent experiments are shown as mean ± SD; *ns*, not significant, ***P < 0.001.

We previously found that TGFβ-induction of the EGFR-dependent invasion-associated genes identified in Fig. 1g and Supplementary Fig. S1d requires AP-1-dependent SMAD2/3 recruitment [25, 32]. We therefore next examined the effects of EGF on TGFβ induction of AP-1 components. As shown in Fig. 3a, b and Supplementary Fig. S3a, b, both the basal and TGFβ-induced levels of JUN, JUNB, FOSL1 and/or FOS, FOSB, FOSL2 were strongly enhanced in the presence of EGF in MCF10A MII, HCC1937, HCC1954 and HCC202 cells. We also analyzed the chromatin binding of the two key AP-1 components JUNB and FOSL1 in cells treated with the combination of EGF and TGFβ vs TGFβ only. As shown in Supplementary Fig. S3c, d, combined EGF and TGFβ treatment resulted in increased binding of JUNB and/or FOSL1 to the SMAD binding regions of *LAMB3*, *WNT7B*, and *ITGA2*.

When analyzed on the mRNA level the differences in TGFβ inducibility between the JUN and FOS family members were striking. In the absence of EGF, both *JUN* and *JUNB* were efficiently induced by 1.5 h treatment with TGFβ, but *FOS, FOSB* and *FOSL1* were not. However, EGF increased the basal mRNA levels of *FOS* and *FOSL1* (Fig. 3c). Interestingly, ChIP-qPCR analysis showed that EGF strongly enhanced TGFβ-induced binding of SMAD2/3 to the *FOS* and *FOSB* loci, while having much less effect on the TGFβ-induced binding of SMAD2/3 to the *JUNB* gene (Supplementary Fig. S3e).

To further examine the regulation of AP-1 by TGFβ and EGF signaling, we compared the effects of TGFβRI, EGFR, MEK, AKT and PI3K inhibition. Inhibition of TGFβRI counteracted the induction by 6 h of TGFβ treatment of the AP-1 components FOS, FOSB, FOSL2, JUN and JUNB, in the presence of EGF (Fig. 3d), which is in line with our previous findings [25]. EGFR inhibition by lapatinib counteracted the TGFβ-induced effects on these proteins as well, and, strikingly, reduced the levels of FOS and FOSL1 even below their basal levels. The MEK inhibitor suppressed the levels of the four FOS family members more efficiently than lapatinib, similar to their effects on phospho-ERK in Fig. 2b. MEK inhibition also completely blocked TGFβ-induction of JUNB, but only had a weak suppressing effect on JUN. In contrast, inhibition of PI3K and AKT did not reduce the TGFβ-induced effects on JUNB and the FOS family, but like the MEK inhibitor partially reduced the levels of JUN (Fig. 3d). In line with these data, the EGFR kinase inhibitor strongly reduced the *FOS* and *FOSL1* mRNA levels induced by TGFβ and EGF. The decrease on JUNB mRNA expression by EGFR kinase inhibitor was less pronounced while there was no significant effect on JUN mRNA levels (Fig. 3e). Together, these results indicate that EGFR signaling enables and potentiates induction of AP-1 (JUN/FOS) by TGFβ both at the protein and mRNA level.

### p63 is critical for EGFR-, JUN/FOS- and TGFβ/SMAD-mediated invasion and gene activation

**Fig. 4:**
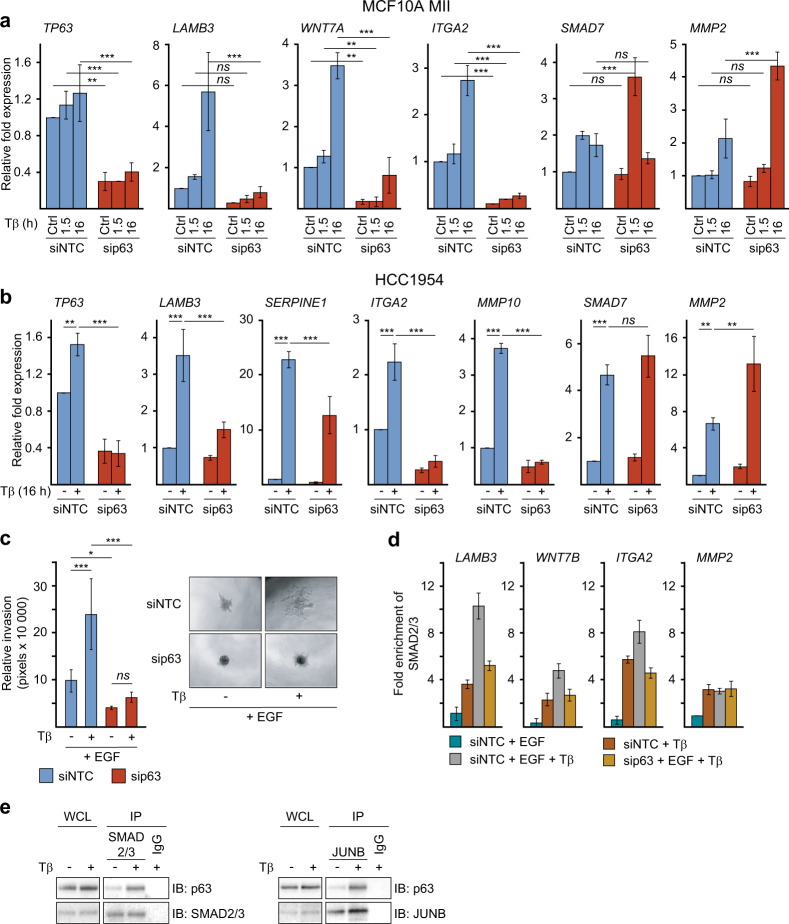
p63 is essential for the pro-invasive SMAD-AP-1 program. **a** qRT-PCR analysis to investigate the role of p63 in TGFβ + EGF-induced gene expression. MCF10A MII cells were transfected with non-targeting control (siNTC) or specific p63 siRNA, serum-starved for 16 h, and stimulated for 1.5 or 16 h with TGFβ1 (5 ng/ml), as indicated. Statistics were calculated using one-way analysis of variance (ANOVA). The data were further analyzed using Tukey’s multiple comparisons test. Results from four independent experiments are shown as mean ± SD; *ns*, not significant, **P < 0.01, ***P < 0.001. **b** qRT-PCR analysis to investigate the effect of p63 depletion on TGFβ + EGF-induced target genes. HCC1954 cells were transfected with non-targeting control (siNTC) or specific p63 siRNA, serum-starved for 16 h, and stimulated for 1.5 (*SMAD7*) or 16 h with TGFβ1 (5 ng/ml), as indicated. Statistics were calculated using one-way analysis of variance (ANOVA). The data were further analyzed using Dunnett’s multiple comparisons test and compared with the results from cells transfected with non-targeting control (siNTC) and treated with TGFβ1 (5 ng/ml). Results from three independent experiments are shown as mean ± SD; **P < 0.01, and ***P < 0.001. **c** Effect of p63 knockdown on collagen-invasion of MCF10A MII spheroids in the presence or absence of 5 ng/ml TGFβ1 and 20 ng/ml EGF, as indicated. Cells were transfected with non-targeting control (siNTC) or specific p63 siRNA before spheroid formation. Left: relative invasion was quantified as the mean area that the spheroids occupied 24 h after being embedded in collagen. Statistics were calculated using one-way analysis of variance (ANOVA). The data were further analyzed using Tukey’s multiple comparisons test. Data represent mean ± SD (*n* ≥ 6 spheroids per condition) and are representative of three independent experiments; *ns*, not significant, *P < 0.05, and ***P < 0.001. Right: representative pictures of spheroids were taken 24 h after embedding. **d** The effect of p63 knockdown on SMAD2/3 recruitment by TGFβ and EGF. ChIP-qPCR showing SMAD2/3 binding to the indicated gene loci in MCF10A MII cells transfected with non-targeting control (siNTC) or specific p63 siRNA, serum-starved with or without EGF (20 ng/ml), and stimulated for 6 h with 5 ng/ml TGFβ1 (5 ng/ml) or untreated, as indicated. One of two independent experiments with similar results, is shown. **e** p63 interaction with SMAD2/3 and JUNB. MCF10A MII cells grown in the presence of EGF (20 ng/ml) were stimulated with TGFβ (5 ng/ml, 45 min) or not, and whole cell lysates (WCL) were immunoprecipitated (IP) with JUNB or SMAD2/3 specific antibodies, or IgG control, and analyzed by immunoblotting. One of three independent experiments with similar results, is shown.

p63 has recently been shown to control epithelial stemness and cell fate specification [34], and its expression has been linked to basal-like breast cancers in correlation with additional basal epithelial markers [33]. Previous work by us and others showed that signaling by EGFR and its ligand HB-EGF can be controlled by p63- and/or JUN-mediated activation of the EGFR and HB-EGF genes [35, 39, 40]. Moreover, co-activation of RAS and TGFβ signaling in HaCaT keratinocytes enhances binding of p63 to its genomic sites via downregulation of mutant p53 [35]. This prompted us to investigate the putative mechanistic involvement of p63 in the TGFβ – EGF cooperation. We first analyzed the role of p63 in MCF10A MII cells, which express wildtype p53, and like HCC1954 and HCC1937 cells, essentially only the ΔNp63α isoform [41–43]. Interestingly, knockdown of p63 strongly reduced the expression of the pro-invasive TGFβ and AP-1 target genes, *LAMB3, WNT7A, ITGA2*, and *MMP10*, whereas p63 knockdown enhanced the expression of *MMP2* and *SNAI1* (Figs. 1g, 4a and Supplementary Fig. S1d and S4a). Similar results were obtained in HCC1954 cells (Fig. 4b) and the parental non-oncogenic MCF10A MI cells, which do not contain active oncogenic RAS (Supplementary Fig. S4b). Knockdown of p63 also suppressed EGF- and TGFβ-dependent invasion of MCF10A MII spheroids in collagen (Fig. 4c). EGF- and TGFβ-induced recruitment of SMAD2/3 to the *LAMB3, WNT7B*, and *ITGA2* gene loci was suppressed by p63 knockdown as well, whereas SMAD2/3 binding to the *MMP2* locus was not affected (Fig. 4d). These results thus strongly suggest that p63 is essential for the pro-invasive SMAD-AP-1 program in these HER2+ and/or EGFR+ breast cancer cells, which may involve binding of p63 to SMAD and AP-1 binding regions [35]. Indeed, endogenous p63 was found to interact with both endogenous SMAD2/3 and JUNB in MCF10A MII cells (Fig. 4e), and EGF treatment was found to increase the binding of p63 to the *LAMB3* and *ITGA2* gene loci (Supplementary Fig. S4c).

**Fig. 5:**
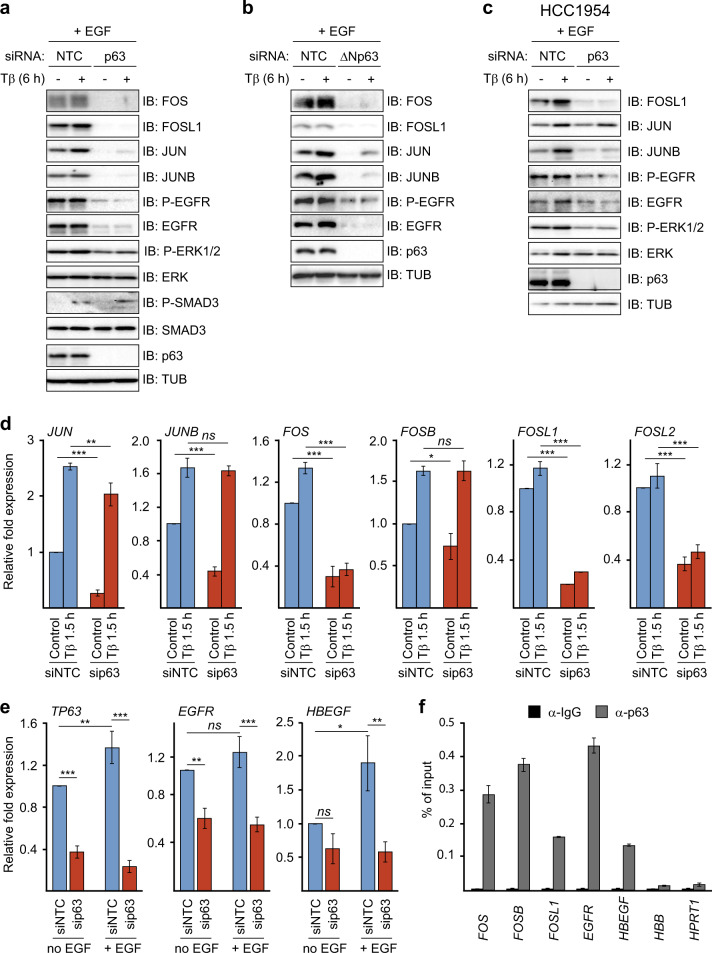
p63 is essential for JUN/FOS, EGFR and HB-EGF expression. The effect of p63 knockdown on TGFβ- and EGF-induced AP-1 and EGFR pathway components. MCF10A MII (**a**, **b**) and HCC1954 (**c**) cells were transfected with non-targeting control (siNTC), p63 siRNA, or ΔNp63-specific siRNA, serum-starved for 16 h, stimulated for 6 h with 5 ng/ml TGFβ1 or untreated, and analyzed by immunoblotting. One of three experiments with similar results, is shown. **d** and **e** MCF10MII cells were treated as in (**a**), stimulated with TGFβ1 (5 ng/ml) for 1.5 h (**d**) or 6 h (**e**), or untreated, and analyzed by qRT-PCR analysis. Statistics were calculated using one-way analysis of variance (ANOVA). The data were further analyzed using Tukey’s multiple comparisons test. Results from three independent experiments are shown as mean ± SD; *ns*, not significant, *P < 0.05, **P < 0.01, and ***P < 0.001. **f** ChIP-qPCR showing p63 binding to the indicated gene loci in MCF10A MII cells. One of two independent experiments with similar results, is shown.

To investigate further the mechanism by which p63 enables and/or enhances activation of the SMAD-AP-1 invasion program by TGFβ and EGF, we analyzed its effect on AP-1 and EGFR pathway components. As shown in Fig. 5a, the levels of TGFβ- and EGF-inducible FOS and JUN members were suppressed upon p63 knockdown, as were the levels of EGFR, auto-phosphorylated EGFR and active phosphorylated ERK1/2 MAPK. In contrast, no effect was observed on the total levels of ERK or on TGFβ-induced phosphorylation of SMAD3. Similar results were obtained upon transfection with ΔNp63-specific siRNA in MII, and MI cells (Fig. 5b and Supplementary Fig. S5a), and in HCC1954 cells (Fig. 5c). mRNA analysis showed that p63 knockdown in particular reduced the levels of *FOS*, *FOSL1*, and *FOSL2* in MII cells (Fig. 5d), and of *FOSL1* in HCC1954 cells (Supplementary Fig. S5b). Knockdown of p63 also reduced the mRNA levels of EGFR and HB-EGF in MCF10A MII cells (Fig. 5e). In line with the putative role of p63 in SMAD and AP-1-induced transcription, p63 was found to bind to the *FOS, FOSB, FOSL1, EGFR* and *HBEGF* gene loci (Fig. 5f). We conclude from these results that p63 can enable and potentiate the EGFR- and AP-1-dependent TGFβ invasion/migration program by activating multiple AP-1 and EGFR pathway components.

**Fig. 6:**
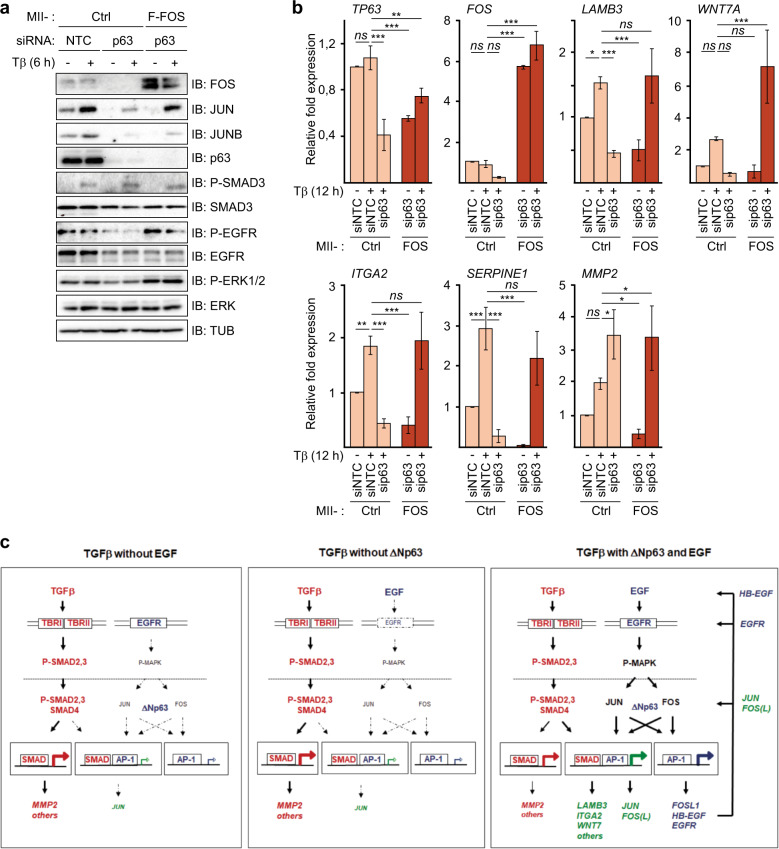
Ectopic overexpression of FOS counteracts the decrease in TGFβ-induced gene activation after p63 depletion. **a**, **b** The effect of ectopic FOS expression on TGFβ- and EGF-induced AP-1 and EGFR pathway components and target genes upon decrease by p63 knockdown. Control or Flag-FOS (F-FOS)-overexpressing MCF10A MII cells were transfected with non-targeting control (siNTC) or p63 siRNA, serum-starved with EGF for 16 h, stimulated with 5 ng/ml TGFβ1 as indicated, or untreated, and analyzed by immnoblotting (**a**), and qRT-PCR analysis (**b**). Statistics were calculated using one-way analysis of variance (ANOVA). The data were further analyzed using Dunnett’s multiple comparisons test and compared with the results from control cells transfected with non-targeting control (siNTC) and treated with TGFβ1 (5 ng/ml). Results from three independent experiments are shown as mean ± SD; *ns*, not significant, *P < 0.05, **P < 0.01, and ***P < 0.001 (**c**) Schematic representation of the distinct (red, blue) and cooperative (green) contributions of TGFβ, SMADs, EGF, JUN, FOS members and p63 to the combined EGF + TGFβ invasion program analyzed in this study. In the absence of EGF (left panel) or ΔNp63 (middle panel) TGFβ (red pathway) mainly induces TGFβ target genes controlled by SMAD sites, such as MMP2, via activation of membrane-localized TGFβRI (TBRI) and TGFβRII (TBRII) which phosphorylate and activate SMAD2 and SMAD3 to bind to SMAD4. On the other arm ΔNp63 and EGF (blue pathway) enable potent levels of EGF signaling via cell-membrane-localized EGFR, which triggers phosphorylation and activation of, amongst others, MAP kinases, which in the nucleus activate and induce JUN and FOS family members, that bind ΔNp63 and subsequently can auto-regulate their own expression via AP-1 sites, and induce *EGFR* and *HB-EGF* (right panel). The combined action of TGFβ and EGF results in enhanced levels of AP-1 and enables and/or potentiates activation of SMAD/AP-1 target genes such as *LAMB3*, *ITGA2* and *WNT7* (green).

Because both p63 and EGFR strongly enhanced the basal and TGFβ-induced levels of FOS mRNA and protein, which can stabilize JUN members [19, 20], we next examined whether ectopic overexpression of FOS can bypass the requirement of p63 in MCF10A MII cells. Indeed, MII cells stably infected with a Flag-FOS lentiviral vector carried increased levels of phosphorylated active EGFR and ERK compared with control MII cells upon p63 knockdown, and also contained higher levels of TGFβ-induced JUN and JUNB (Fig. 6a). Moreover, ectopic overexpression of FOS counteracted the decrease in the TGFβ-induced mRNA levels of *LAMB3*, *WNT7A*, *ITGA2* and *SERPINE1* after p63 depletion (Fig. 6b).

In summary, these results show that ΔNp63 is necessary for the activation of the EGFR-, and AP-1-dependent invasion gene program induced by TGFβ in multiple breast cancer cell lines. Moreover, by enhancing the levels of EGFR, HB-EGF, JUN and, in particular, FOS family members, ΔNp63 can enable sustained activation of the pro-oncogenic gene program induced by SMADs and AP-1 in HER2+ and/or EGFR+ breast cancer cells.

## Discussion

TGFβ has a biphasic role in breast tumor progression [7, 13]. In the early stages, TGFβ-SMAD signaling inhibits cell growth and thus acts as a tumor suppressor. In late stage tumors, TGFβ usually functions as a tumor promoter, e.g., by stimulating EMT, i.e., trans-differentiation of epithelial cells to cells with more mesenchymal characteristics, and invasive and metastatic potential. These tumor cells have escaped TGFβ-induced growth inhibitory and apoptotic responses, but have retained or gained certain other responses to TGFβ stimulation. In various cell types, TGFβ requires oncogenic RAS signaling to efficiently induce an EMT program [4, 5]. Here we have shown that the TGFβ and EGFR pathways cooperate to activate an AP-1- and p63-dependent invasion program in various HER2+ and/or EGFR+ breast cancer cell lines. Moreover, by enhancing the levels of EGFR, HB-EGF, JUN and, in particular, FOS family members, ΔNp63 can promote the pro-oncogenic transcriptional program of SMAD and AP-1 in breast cancer cells. We thus identified an important mechanism by which oncogenic changes and environmental changes in breast tumors can re-direct TGFβ-SMAD signaling towards tumor progression. This might help in the design of appropriate combination therapies, since clinical inhibitors of TGFβ might either inhibit or enhance tumor progression, depending on other oncogenic defects and genetic background. Cross-talk between EGFR and TGFβ signaling is possible at multiple levels, e.g., by post-translational regulation of the SMAD proteins by AKT and ERK [4, 5, 13, 14] and by induction of TGFβ1 and HB-EGF [9]. Based on our experiments with specific chemical inhibitors the MEK-ERK pathway appeared to be most critical for the pro-invasive cooperation between EGF and TGFβ in MCF10A-MII cells, and for the activation of AP-1. However, SMAD3-Ser208 phosphorylation by AKT appears to be critical in other cell types [14]. Interestingly, in basal-like and mesenchymal breast cancers the PI3K-AKT and MEK-ERK pathways are often activated, resulting in high levels of JUN and FOSL1, whereas luminal A breast cancers do not show activation of the ERK1/2 MAP-kinase pathway [44–47]. Luminal and estrogen-responsive breast cancers express low levels of AP-1 [23, 48] and appear to respond to TGFβ only weakly; however, not all mesenchymal breast cancers express high levels of p63. Moreover, EGFR signaling appears to exert different roles in breast cancer cells during invasion of primary tumors, dissemination and metastasis [49].

In the HER2+/EGFR+ breast cancer cell lines examined here, EGF by itself already induced high levels of AP-1 protein, but only low levels of invasion related gene expression and subsequent cell invasion. On the other hand, TGFβ/SMAD signaling by itself was not sufficient. In the model in Fig. 6c, we have schematically depicted the distinct and cooperative contributions of TGFβ, SMADs, EGF, JUN, FOS and p63 to the invasion program. It should be noted that autocrine TGFβ signaling to some extent might contribute to the effects of EGF alone. We also would like to stress that some of the TGFβ effects on AP-1 appear to be largely EGF-independent, e.g., TGFβ—induction of JUN and JUNB mRNA and protein in MII cells (Fig. 3a, b). However, we found that the synergism between EGF and TGFβ pathways is important for efficient recruitment of JUNB, FOSL1 and p63 to the promoter regions of genes involved in the late TGFβ-induced invasion program such as *LAMB3* and *ITGA2*.

The mechanism by which p63 enhances SMAD- and AP-1-dependent gene expression remains to be further elucidated. We found that ΔNp63 enhances EGFR-ERK signaling and AP-1 levels by activating the *EGFR* and *HBEGF* genes, directly binding to various *FOS* family gene loci and activating their transcription, and interacting with both SMAD2/3 and JUNB proteins. p63 might thus stabilize the formation of a complex between SMAD and AP-1 or enhance complex formation on the chromatin. However, the role of the α, β, γ, and ε isoforms of ΔNp63 is still unclear. In fact, expression of ΔNp63α has been found to be suppressed by oncogenic PI3K, AKT and RAS [50]. It should further be noted that the p63 family members p53 and p73 also have been found to functionally and physically interact with specific AP-1 components [20, 51, 52].

Finally, our results on TGFβ-SMAD and p63-EGFR-AP-1 cooperation may be relevant for the reported inhibitory effects of TGFβ on the cellular response to anti-cancer drugs. The mechanisms of EMT-associated, TGFβ-induced drug-resistance, are not known, but EGFR, MEK-ERK and AP-1 are likely to be involved [29, 53–59].

In summary, we have identified specific oncogenic functions of the TGFβ-SMAD, EGFR, and p63 pathways in EMT and invasion of HER2+ and/or EGFR+ breast cancer cells. These functions are of importance for future personalized cancer therapeutic strategies, in particular for patients with tumors with ΔNp63 expression.

## Materials and methods

### Cell culture

MCF10A MI and MII cells were obtained from Dr Fred Miller (Barbara Ann Karmanos Cancer Institute, Detroit, USA) and maintained at 37 °C and 5% CO_2_ in DMEM/F12 (Gibco), supplemented with 5% fetal bovine serum (FBS) (Biowest), 20 ng/ml EGF (PeproTech), 100 ng/ml cholera toxin (Sigma-Aldrich), 0.5 µg/ml hydrocortisone (Sigma-Aldrich), 10 µg/ml insulin (Sigma-Aldrich) (complete medium). Cells were starved in DMEM/F12 supplemented with 0.2% FBS, 100 ng/ml cholera toxin, 0.5 µg/ml hydrocortisone, and 10 µg/ml insulin, with or without 20 ng/ml EGF for 16 h prior to TGFβ treatment. HCC1954 breast cancer cells (obtained from Dr Andrew J. G. Simpson, Ludwig Cancer Research, New Your, USA), HCC1937 and HCC202 breast cancer cells (obtained from SE Le Dévédec, Leiden Academic Center for Drug Research, Leiden, the Netherlands) were maintained in RPMI-1640 (Sigma-Aldrich), supplemented with 10% FBS (Biowest). Cells were kept in 0.2% FBS starvation media with or without EGF (20 ng/ml) for 16 h prior to TGFβ treatment. The cell lines were frequently tested for absence of mycoplasma and were authenticated by identity testing.

A detailed description of the materials and methods, including the primer sequences used for qRT-PCR (Table S1) and ChIP-qPCR (Table S2), used in this study is available in the online Supplementary Material and Methods.

## Supplementary information

Supplementary Information
