# Supplementary material for: TGFβ and EGF signaling orchestrates the AP-1- and p63 transcriptional regulation of breast cancer invasiveness
Source: Oncogene. 2020 Apr 29;39(22):4436–49. doi: 10.1038/s41388-020-1299-z (PMC7253358; doi:10.1038/s41388-020-1299-z)
Supplement: Supplementary file 1 — Supplementary Information [file 41388_2020_1299_MOESM1_ESM.doc]

**Supplementary Information**

**TGFβ and EGF signaling orchestrates the AP-1- and p63 transcriptional regulation of breast cancer invasiveness**

Anders Sundqvist^1^,*, Eleftheria Vasilaki^1^, Oleksandr Voytyuk^1^, Yu Bai^1^, Masato Morikawa^2^, Aristidis Moustakas^1^, Kohei Miyazono^1,2^, Carl-Henrik Heldin^1^*,* Peter ten Dijke^1,3^

and Hans van Dam^3^,*

**Material and Methods**

**Supplementary Figures and Tables**

**Material and Methods**

# Lentiviral transduction

MCF10A MII cells were infected with a lentivirus encoding a FLAG-tagged version of human FOS ^59^. As control an empty pLKO vector was used. Virus transduction was performed overnight and the infected cells were selected using culture medium containing puromycin.

# Reagents and antibodies

Recombinant human TGFβ1 and EGF were from PeproTech. The following kinase inhibitors were used at the indicated concentrations: TGFβ type I kinase inhibitors (ALK5i) SB505124 (2.5 µM; Sigma-Aldrich) and LY364947 (2.0 µM; Calbiochem), EGFR inhibitor (EGFRi) lapatinib (10 µM), MEK1 inhibitors (MEKi) PD184352 (0.5 µM: Sigma-Aldrich) and AZD6244 (0.25 µM; Selleckchem), PI3K inhibitor (PI3Ki) LY294002 (10 µM; Calbiochem), and the AKT inhibitor (AKTi) MK-2206 (2.5 µM; Selleckchem). All kinase inhibitors were dissolved in DMSO. Puromycin was purchased from Invitrogen and used at a concentration of 0.5 µg/ml.

The ΔNp63 specific On-target plus SMARTpool siRNA (sense sequence: GGACAGCAGCAUUGAUCAAUU, antisense sequence: 5´-PUUGAUCAAUGCUGCU

GUCCUU) and the On-target plus Non-targeting Control siRNA (D-001810-01-20) were from Dharmacon (Thermo Fisher Scientific). Stealth duplex siRNAs specific for p63 (Cat No.HSS189462) and control siRNA (Cat No. 12935-300) were from Invitrogen (Life Technologies). siRNAs were transfected using siLentFect (Bio-Rad) transfection reagents according to manufacturer’s instructions at 25 nM final concentration.

Antibodies against the following proteins were used: AKT (#4691, Cell Signaling Technology), phospho-Thr308-AKT (#4056, Cell Signaling Technology), phospho-Ser473-AKT (#9271, Cell Signaling Technology), EGFR (#2232, Cell Signaling Technology), phospho-Tyr1068-EGFR (#3777, Cell Signaling Technology), ERK1/2 (#4695, Cell Signaling Technology), phospho-Thr202/Tyr204 ERK1/2 (#4370, Cell Signaling Technology), JUN (#610327, BD Transduction Laboratories), JUNB (sc-8051, Santa Cruz), FN1 (F3648, Sigma-Aldrich), FOS (sc-52, Santa Cruz), FOSB (#2251, Cell Signaling Technology), FOSL1 (sc-22794, Santa Cruz), FOSL2 (sc-604, Santa Cruz), p63α (sc-8344, Santa Cruz, used for western blot analysis), p63 (ab124762, Abcam, used for ChIP experiments), phospho-Thr389-p70 S6K (#9205, Cell Signaling Technology), SERPINE1 (#612024, BD Transduction Laboratories), SMAD2/3 (#610843, BD Transduction Laboratories), phospho-Ser465/467 SMAD2 (#3108, Cell Signaling Technology), SMAD3 (#9523, Cell Signaling Technology and ab28379, Abcam), phospho-Ser423/425 SMAD3 (#9520, Cell Signaling Technology), SMAD4 (sc-7966, Santa Cruz), and TUB (T0198, Sigma-Aldrich).

# 3D spheroid collagen invasion assays

Spheroid invasion into collagen was performed as described previously ^25,32,36^. Briefly, one thousand MCF10A MII or HCC1937 cells were trypsinized, re-suspended in complete medium, or complete medium lacking EGF, supplemented with 2.4 mg/ml methylcellulose (Sigma-Aldrich) and added into each well of a U-bottom 96-well-plate (Greiner Bio One) allowing the formation of one spheroid per well. For siRNA-mediated knockdown, the trypsinized cells were transfected with control or p63 specific siRNA using siLentFect (BioRad) transfection reagent according to manufacturer’s instructions at 50 nM final concentration. Two days after plating, single spheroids were embedded in a 1:1 mix of neutralized Collagen-I (PureCol, Advanced BioMatrix) and complete medium, or complete medium lacking EGF, supplemented with 12 mg/ml of methylcellulose, and allowed to polymerize on the top of neutralized collagen-I in a 96-well-plate. TGFβ1 was directly added to the embedding solution. Kinase inhibitors SB505124, LY394946, lapatinib, PD184352, AZD6244, MK-2206, and LY294002 were added to the medium on the top of the collagen because they were dissolved in DMSO. Pictures were taken at day 0 and day 1 after embedding and quantified by measuring the area occupied by cells using Adobe Photoshop CC 2017 software.

# In vitro wound-healing assay

MCF10A MII cells (3x10^5^ per well) were seeded in a 6-well plate. Medium was changed to starvation medium (complete medium, supplemented with 0.2% FBS, or complete medium without EGF, supplemented with 0.2% FBS) after which cells were grown until ~ 90% confluence. The cell monolayer was wounded by crossed scratching using a 200-μl pipette tip. After washing with phosphate-buffered saline (PBS) the cells were incubated with the indicated agents, i.e. 20 ng/ml EGF, 5 ng/ml TGFβ1, 2.5 µM SB505124, and/or 1 µM lapatinib, and the same crossed scratch wounds were photographed at the indicated time points using an inverted-phase-contrast microscope (Zeiss Axiovert 40CFL). TScratch software was used for quantification of the scratch wound; eight measurements per sample were performed.

# **Western blot analysis**

Cells were seeded in 6-well-plates (2.5 x 10^5^ cells/well). The following day, cells were starved 16 h in complete medium, supplemented with 0.2% FBS, or complete medium without EGF, supplemented with 0.2% FBS. Cells were then stimulated with 20 ng/ml of EGF and/or 5 ng/ml of TGFβ1 for the indicated time-periods. Cells were lysed in 2 x SDS Laemmli sample buffer (5% SDS, 25% glycerol, 150 mM Tris-HCl pH 6.8, 0.01% bromophenol blue, 100 mM dithiothreitol (DTT)). Samples were separated by SDS-PAGE, blotted onto nitrocellulose membrane (Amersham Protran, GE Healthcare Life Science), and the chemiluminescent signal was detected using the Immobilon Western kit (Merck Millipore).

# RNA isolation, cDNA synthesis and quantitative real-time PCR

Total RNA was isolated by Total RNA Purification Kit (Norgen Biotek Corp). cDNA was prepared by using High Capacity cDNA Reverse Transcription Kit (Applied Biosystems) using 0.5 µg of total RNA, according to the manufacturer’s instructions. The cDNA samples were diluted 10 times with water. qRT-PCR was performed using 2x qPCR SyGreen Mix (PCR Biosystems) and BioRad CFX96 real-time PCR detection system according to the manufacturer’s instructions. Relative gene expression was determined using the ΔΔCt method. The expression was normalized to the *GAPDH* gene and quantified relative to the control condition. The complete primer list can be found in Table S1 in the Supplementary Information.

# Chromatin immunoprecipitation (ChIP) and co-immunoprecipitation

ChIP was performed as previously described in ^32,35^. Briefly, cells were fixed in 1% formaldehyde, washed with ice-cold PBS, harvested by scraping, pelleted, and re-suspended in 1 ml of SDS lysis buffer (1% SDS, 50 mM Tris-HCl, pH 8.0, 10 mM EDTA, supplemented with protease inhibitors (Complete EDTA-free protease inhibitors; Roche Diagnostics)). Samples were sonicated three times and centrifuged at 14,000 rpm at 4°C for 10 min. After removal of a control aliquot (whole-cell extract), supernatants were diluted in ChIP dilution buffer (1% Triton X-100, 20 mM Tris-HCl, pH 8.0, 150 mM NaCl, 2 mM EDTA), and incubated at 4°C overnight with antibodies coupled to anti-mouse IgG-Dynabeads (Invitrogen) in PBS with 0.5% bovine serum albumin. The beads were washed five times in ChIP washing buffer (50 mM HEPES-KOH, pH 7.0, 0.5 M LiCl, 1 mM EDTA, 0.7% deoxycholate, 1% Igepal CA630) and once with TE buffer (10 mM Tris-HCl, pH 8.0, 1 mM EDTA). Immunoprecipitated samples were eluted and reverse cross-linked at 65°C in SDS lysis buffer. Genomic DNA was extracted with a PCR purification kit (Qiagen). The immunoprecipitated DNA was analyzed by qRT-PCR using locus specific primers (the complete primers list can be found in Table S2 in the Supplementary Information) and normalized to the input DNA. Relative fold enrichment corresponded to the specific enrichment in each gene locus divided by the enrichment in the negative control regions (*hemoglobin β* (*HBB)* promoter and *HPRT1* first intron) and quantified relative to the control- or the siNTC-condition as indicated.

For the co-immunoprecipitation assay, MCF10A MII cells treated with 5 ng/ml of TGFβ1 for 45 min were lysed in lysis buffer (1% Triton X-100, 20 mM Tris-HCl, pH 7.5, 150 mM NaCl, 10% glycerol) and incubated overnight with anti-mouse IgG Dynabeads (Life Technologies) that had been preincubated with SMAD2/3 or JUNB antibody or IgG control in PBS, supplemented with 0.5% bovine serum albumin. The beads were washed three times with lysis buffer and the immunoprecipitated proteins were eluted in 2x SDS sample buffer and subjected to SDS-PAGE.

# Statistical analysis

Collagen invasion assays contained n ≥ 6 spheroids for each condition, and was repeated at least twice with similar results. For migration assays and qRT-PCR, at least three independent experiments were performed. The statistical difference between indicated experimental groups were determined by one-way analysis of variance (ANOVA). Given a significant overall effect of treatment (*P*<0.05), further analysis was carried out using Tukey’s or Dunnett’s multiple comparisons test with **P*<0.05, ***P*<0.01, and ****P*<0.001 being considered significant. Data are presented as mean ± SD.

**Supplementary Figures and Tables**


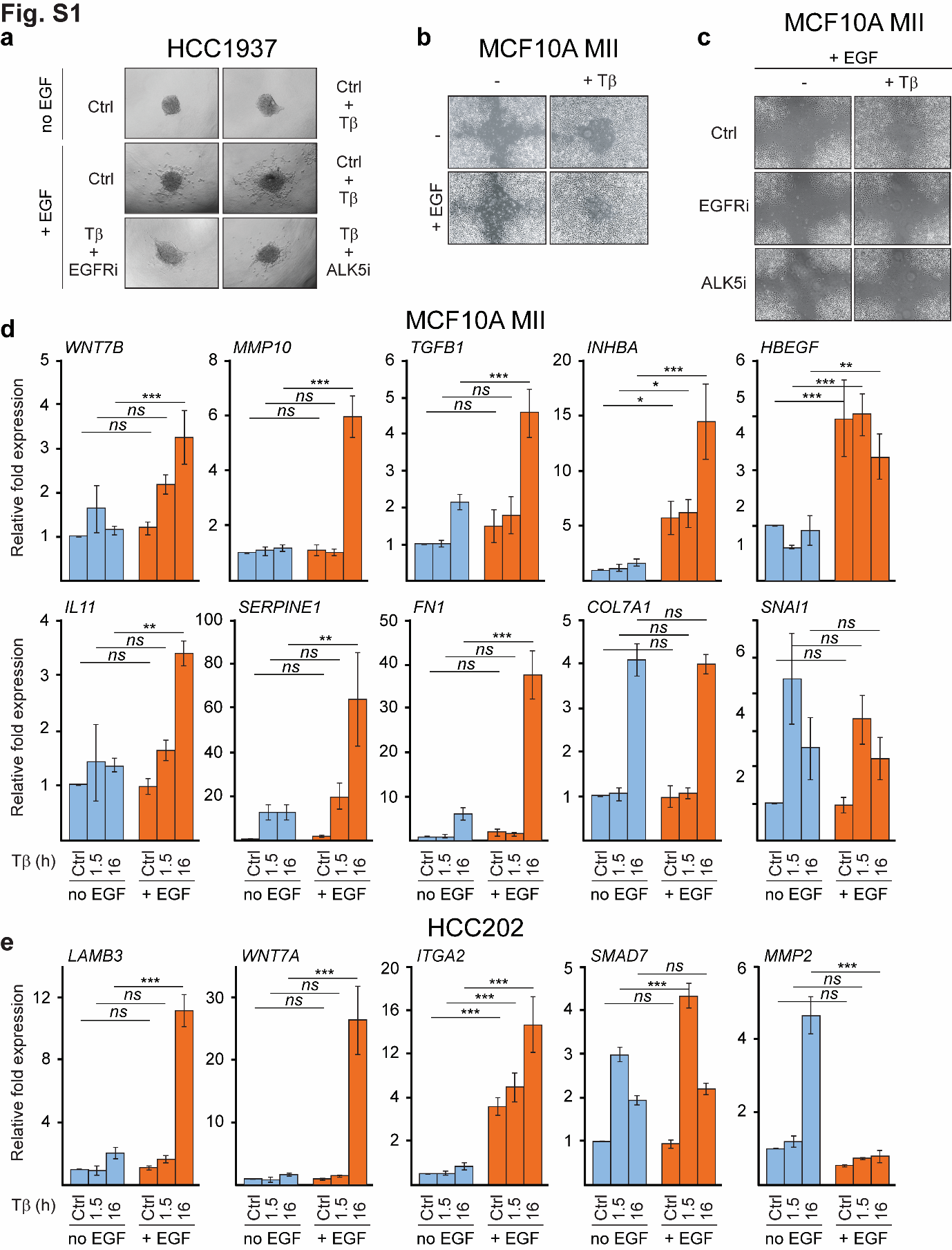


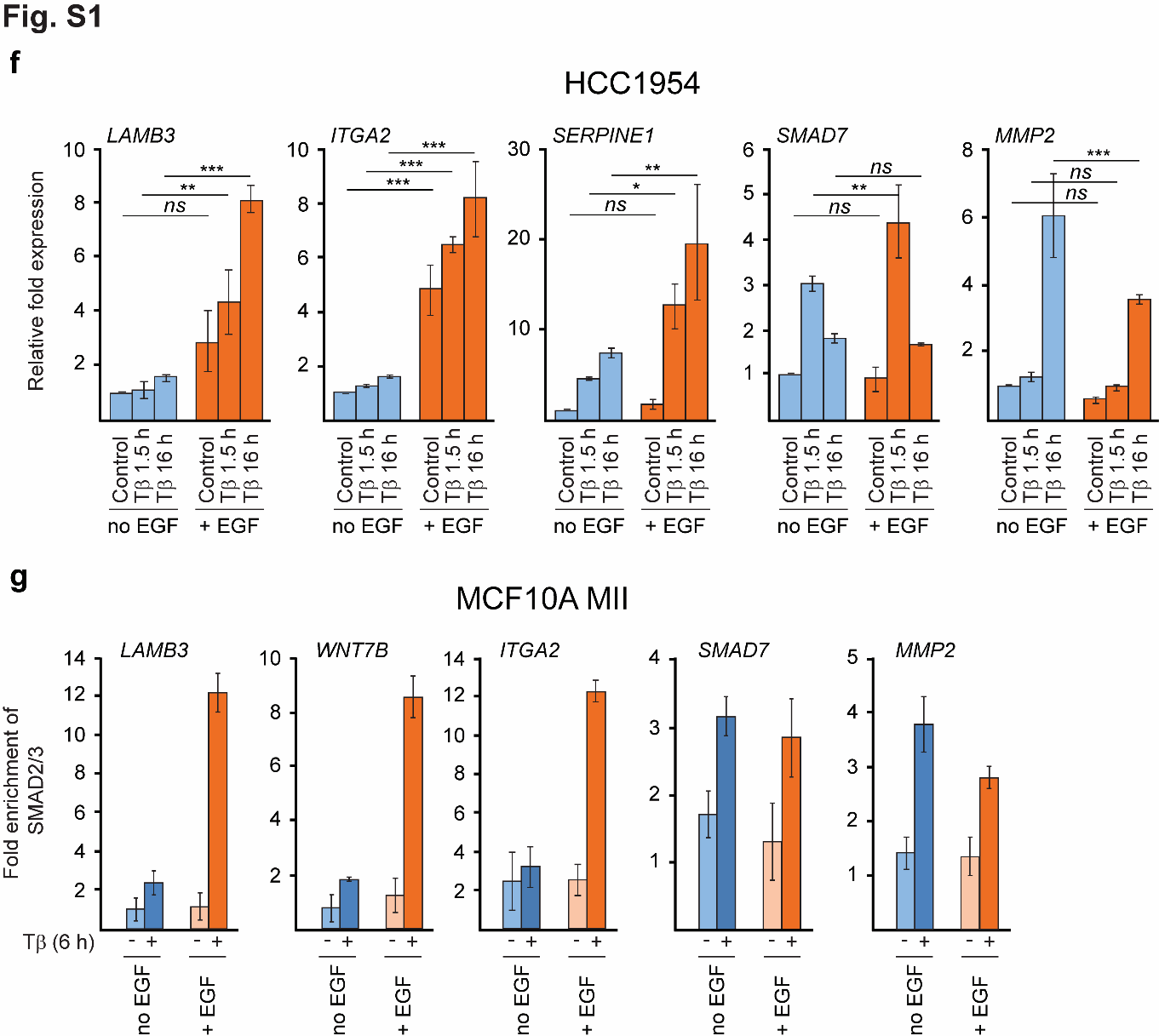


**Figure S1. Effects of TGFβ and EGF- on TGFβ-induced EMT and invasion-associated genes.** **(a)** Collagen-invasion of HCC1937 spheroids in the presence or absence of TGFβ1 (5 ng/ml), EGF (20ng/ml), 10 µM lapatinib (EGFRi) and 2.5 µM SB505124 (ALK5i) as indicated. Representative pictures of spheroids were taken 28 h after embedding are shown. **(b-c)** Migration of MCF10A MII cells in the presence or absence of TGFβ (5 ng/ml) and EGF (20 ng/ml), (1 µM) lapatinib (EGFRi) and (2.5 µM) SB505124 (ALK5i) as indicated measured by wound healing (scratch) assays. Representative pictures of the cells 48h after scratching are shown. (**d-f**) MCF10A MII (**d**) cells were incubated for 16 h in starvation medium (EGF, insulin, cholera toxin, hydrocortisone, 0.2 % FBS), or in starvation medium lacking EGF, and subsequently treated with 5 ng/ml TGFβ1 for 0, 1.5 or 16 h, as indicated. HCC202 (**e**) and HCC1954 (**f**) cells were incubated in starvation medium (0.2 % FBS and EGF (20 ng/ml)), or in starvation medium lacking EGF, and subsequently treated with 5 ng/ml TGFβ1 for 0, 1.5 or 16 h, as indicated. mRNA levels were analyzed by qRT-PCR analysis. Statistics were calculated using one-way analysis of variance (ANOVA). The data were further analyzed using Tukey’s multiple comparisons test. Results from four independent experiments are shown as mean ± SD; *ns*, not significant, **P*<0.05, ***P*<0.01, and ****P*<0.001. (**g**) ChIP-qPCR showing SMAD2/3 binding to the indicated gene loci in MCF10A MII cells serum-starved with or without EGF (20 ng/ml) as in (**d**), and stimulated for 6 h with TGFβ1 (5 ng/ml) or untreated, as indicated. One of two independent experiments with similar results, is shown.


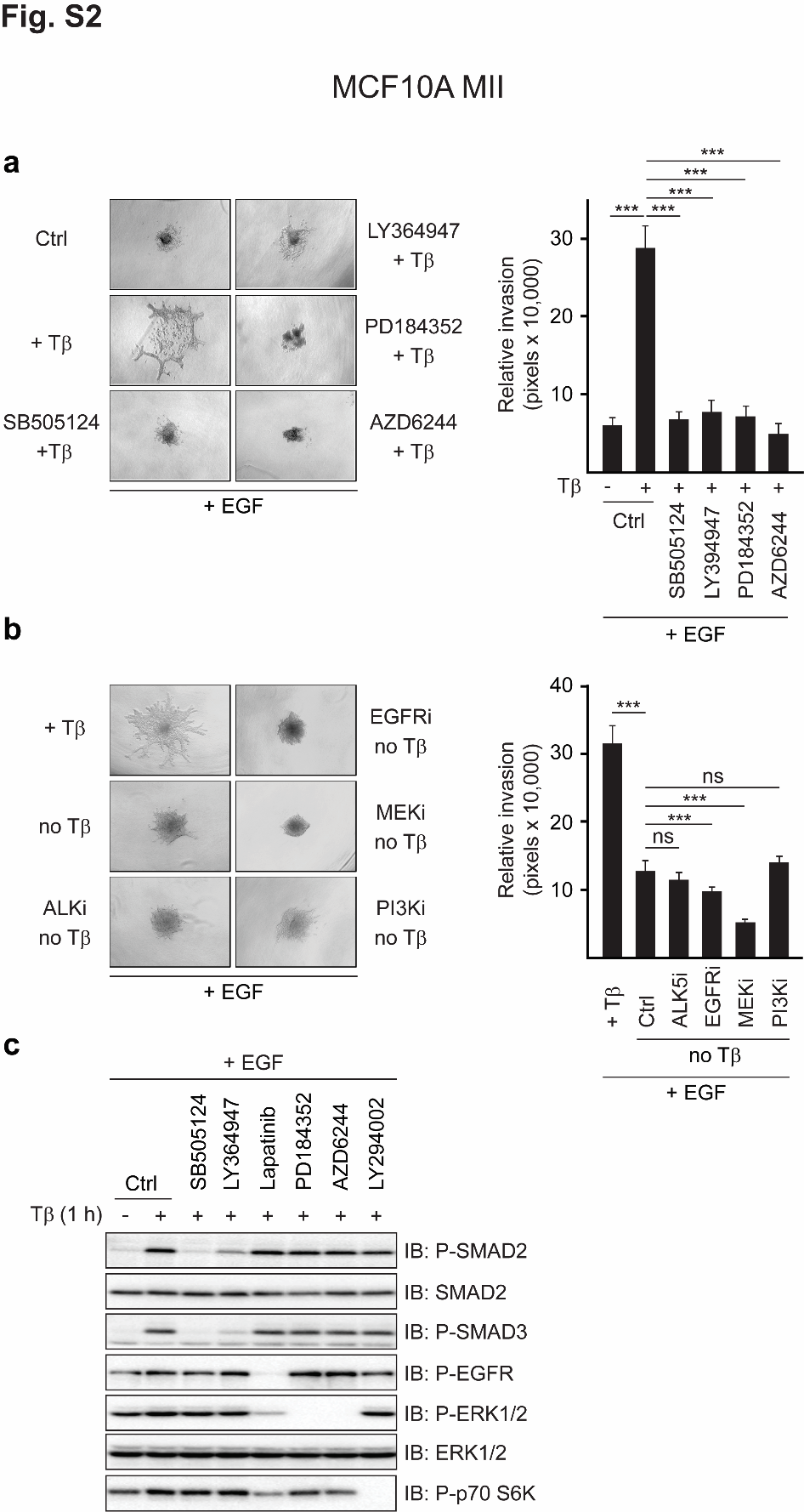


**Figure S2**. **Effect of inhibition of EGFR, MEK and PI3K on MCF10A MII cell invasion in the presence and absence of TGFβ1.** (**a**) Comparison of 2.5 µM SB505124 (ALK5i), 2.0 µM LY364947 (ALK5i), 0.5 µM PD184352 (MEKi), and 0.25 µM AZD6244 (MEKi) on collagen-invasion of MCF10A MII spheroids in the presence of EGF (20 ng/ml). Left: representative pictures of spheroids were taken 40 h after embedding. Right: relative invasion was quantified as the mean area that the spheroids occupied 40 h after being embedded in collagen. (**b**) The effects of the kinase inhibitors 2.5 µM SB505124 (ALK5i), 10 µM lapatinib (EGFRi), 0.25 µM AZD6244 (MEKi), and 10 µM LY294002 (PI3Ki) on collagen-invasion of MCF10A MII spheroids in the presence of EGF (20 ng/ml). Left: representative pictures of spheroids were taken 28 h after embedding. Right: relative invasion was quantified as the mean area that the spheroids occupied 28 h after being embedded in collagen. Statistics were calculated using one-way analysis of variance (ANOVA). The data were further analyzed using Dunnett’s multiple comparisons test and compared with the results from cells treated with TGFβ1 (5 ng/ml) alone (ctrl +Tβ) **(a)** or control without TGFβ1 (ctrl no Tβ) **(b)**. Data represent mean ± SD (*n* ≥ 6 spheroids per condition) and are representative of three independent experiments; *ns*, not significant, ****P*<0.001. (**c**) Immunoblot validation of kinase inhibitor specificity. Comparison of 2.5 µM SB505124 (ALK5i), 2.0 µM LY364947 (ALK5i), 10 µM lapatinib (EGFRi), 2.0 µM PD184352 (MEKi), 0.25 µM AZD6244 (MEKi), and 10 µM LY294002 (PI3Ki). MCF10A MII cells were incubated for 16 h in 0.2% FBS starvation medium with EGF (20 ng/ml) before addition of the indicated kinase inhibitors or DMSO (control). 15 min later TGFβ1 (5 ng/ml) was added and incubation prolonged for 1 h. One of three independent experiments with similar results, is shown.


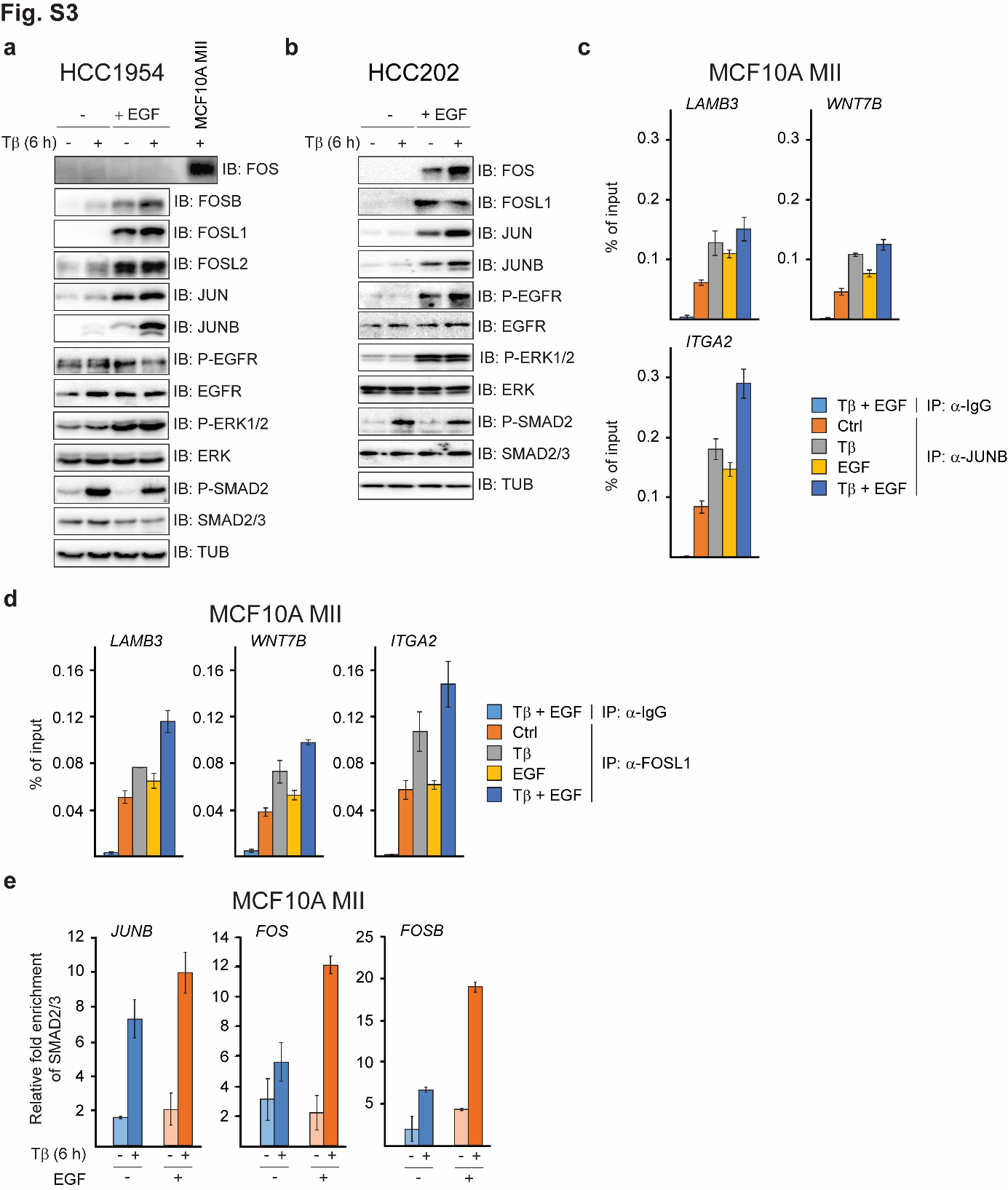


**Figure S3.** **EGFR-MEK signaling enables and/or potentiates TGFβ induction of AP-1 components.** **(a-b)** HCC1954 **(a)** or HCC202 **(b)** cells were incubated for 16 h in starvation medium (0.2 % FBS and EGF (20 ng/ml)), or in starvation medium lacking EGF, subsequently treated with 5 ng/ml TGFβ1 for 0 or 6 h, as indicated, and analyzed by immunoblotting. One of three independent experiments with similar results, is shown. **(c-e)** The effect of EGF and TGFβ treatment on AP1 and SMAD2/3 recruitment to genomic regions. ChIP-qPCR showing binding of JUNB (**c**) FOSL1 (**d**) and SMAD2/3 (**e**) to the indicated gene loci in MCF10A MII cells that were serum-starved in the presence or not of EGF (20 ng/ml), and stimulated for 6 h with 5 ng/ml TGFβ1 or untreated, as indicated. One representative of three independent experiments with similar results, is shown.


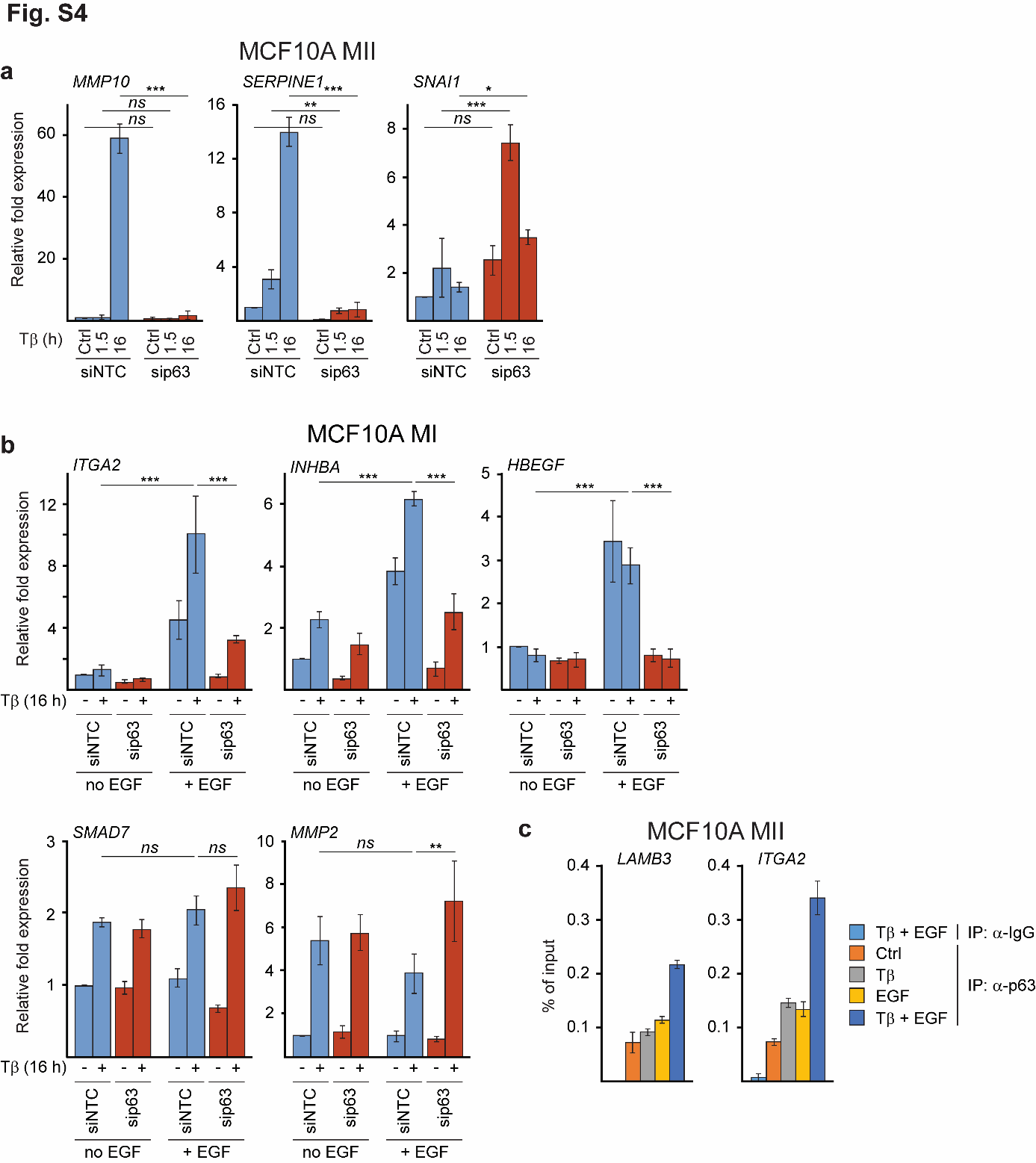


**Figure S4. p63 is essential for the pro-invasive SMAD-AP-1 program.** **(a)** qRT-PCR analysis to investigate the role of p63 in TGFβ+EGF-induced gene expression. MCF10A MII cells were transfected with non-targeting control (siNTC) or specific p63 siRNA, serum-starved for 16 h, and stimulated for 1.5 or 16 h with TGFβ1, as indicated. Statistics were calculated using one-way analysis of variance (ANOVA). The data were further analyzed using Tukey’s multiple comparisons test. Results from four independent experiments are shown as mean ± SD; *ns*, not significant, **P*<0.05, ***P*<0.01, and ****P*<0.001. **(b)** MCF10A MI cells were transfected with non-targeting control (siNTC) or specific p63 siRNA, incubated for 16 h in 0.2% FBS starvation medium with or without EGF (20 ng/ml) and subsequently treated with TGFβ1 (5 ng/ml) for 16 h. Statistics were calculated using one-way analysis of variance (ANOVA). The data were further analyzed using Dunnett’s multiple comparisons test and compared with the results from cells transfected with non-targeting control (siNTC) and treated with TGFβ1 in the presence of EGF. Results from three independent experiments are shown as mean ± SD; *ns*, not significant, ***P*<0.01 ****P*<0.001. **(c)** The effect of EGF and TGFβ treatment on p63 recruitment to genomic regions. ChIP-qPCR showing binding of p63 to the indicated gene loci in MCF10A MII cells that were serum-starved in the presence or not of EGF (20 ng/ml), and stimulated for 6 h with 5 ng/ml TGFβ1 or untreated, as indicated. One representative of three independent experiments with similar results, is shown.


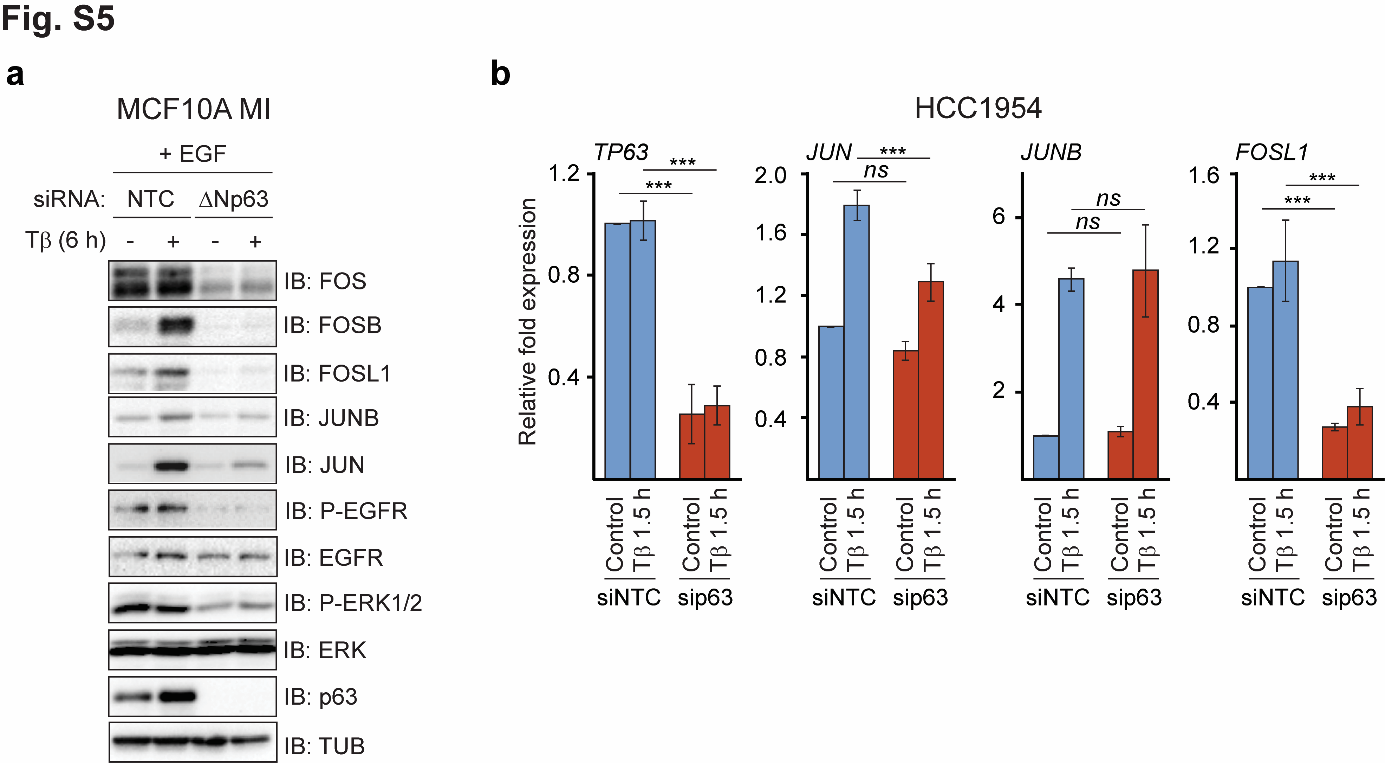


**Figure S5. p63 is essential for JUN/FOS and EGFR expression.** (**a**) MCF10A MI cells were transfected with non-targeting control (siNTC) or ΔNp63 specific siRNA, serum-starved for 16 h, stimulated with TGFβ1 (5 ng/ml) as indicated and analyzed by immunoblotting. One of three independent experiments with similar results, is shown. **(b)** HCC1954 cells were transfected with non-targeting control (siNTC) or p63 siRNA, serum-starved for 16 h, stimulated or not for 1.5 h with 5 ng/ml TGFβ1 and analyzed by qRT-PCR analysis. Statistics were calculated using one-way analysis of variance (ANOVA). The data were further analyzed using Tukey’s multiple comparisons test. Results from three independent experiments are shown as mean ± SD; *ns*, not significant, ****P*<0.001.

| **Primer sequences for qRT-PCR** | |
| --- | --- |
| **Name** | **Sequence** |
| ***COL7A1* FW** | 5'-CTGCACTTGTCGGGGACCGC-3' |
| ***COL7A1* Rev** | 5'-AGCCTCAGGCGGCGTAGTGA-3' |
| ***EGFR* FW** | 5'-GGCACTTTTGAAGATCATTTTCTC-3' |
| ***EGFR* Rev** | 5'-CTGTGTTGAGGGCAATGAG-3' |
| ***FOS* FW** | 5'-CTGGCGTTGTGAAGACCAT-3' |
| ***FOS* Rev** | 5'-TCCCTTCGGATTCCTTTT-3' |
| ***FOSB* transcript variant 1 FW** | 5'-TCACCCCAGAGGAAGAGGAG-3' |
| ***FOSB* transcript variant 1 Rev** | 5'-AACTGATCTGTCTCCGCCTG-3' |
| ***FOSL1* FW** | 5'-CTCTTCCTGTGATCCACCCAA-3' |
| ***FOSL1* Rev** | 5'-TGGGTAAAGTGGCACCTTCTG-3' |
| ***FOSL2* FW** | 5'-ATCCCGGGAACTTTGACACC-3' |
| ***FOSL2* Rev** | 5'-TACCCGGAATTTCTGCTGGC-3' |
| ***GAPDH* FW** | 5'-GGAGTCAACGGATTTGGTCGTA-3' |
| ***GAPDH* Rev** | 5'-GGCAACAATATCCACTTTACCA-3' |
| ***HBEGF* FW** | 5'-TCCTCTCGGTGCGGGACCAT-3' |
| ***HBEGF* Rev** | 5'-GTGCCGAGAGAACTGCAGCCAG-3' |
| ***ITGA2* FW** | 5'-GTCGGTGCTCCTCGGGCAAA-3' |
| ***ITGA2* Rev** | 5'-TGGTCACCTCGGTGAGCCTGA-3' |
| ***JUN* FW** | 5'-GCTAACGCAGCAGTTGCAAAC-3' |
| ***JUN* Rev** | 5'-CCGTCGCAACTTGTCAAGTTC-3' |
| ***JUNB* FW** | 5'-ACTCATACACAGCTACGGGATACG-3' |
| ***JUNB* Rev** | 5'-GGCTCGGTTTCAGGAGTTTG-3' |
| ***LAMB3* FW** | 5'-ACGGCAGAACACACAGCAAGGA-3' |
| ***LAMB3* Rev** | 5'-ACCGGGTCCTCCCAACAAGCA-3' |
| ***MMP2* FW** | 5'-AGATGCCTGGAATGCCAT-3' |
| ***MMP2* Rev** | 5'-GGTTCTCCAGCTTCAGGTAAT-3' |
| ***MMP10* Fw** | 5'-CCCCTGGTGCCCACAAA-3' |
| ***MMP10* Rev** | 5'-TCACACTTGGCTGGCATCTC-3' |
| ***SERPINE1* FW** | 5'-GAGACAGGCAGCTCGGATTC-3' |
| ***SERPINE1* Rev** | 5'-GGCCTCCCAAAGTGCATTAC-3' |
| ***SMAD7* FW** | 5'-ACCCGATGGATTTTCTCAAACC-3' |
| ***SMAD7* Rev** | 5'-GCCAGATAATTCGTTCCCCCT-3' |
| ***SNAI1* FW** | 5'-CACTATGCCGCGCTCTTTC-3' |
| ***SNAI1* Rev** | 5'-GCTGGAAGGTAAACTCTGGATTAGA-3' |
| ***TP63* FW** | 5'-AACCAGAGATGGGCAAGTCCTGGGC-3' |
| ***TP63* Rev** | 5'-ATCCGCCTTCCTGTCTCTTCCTGGG-3' |
| ***WNT7A* FW** | 5'-TGCCCGGACTCTCATGAAC-3' |
| ***WNT7A* Rev** | 5'-GTGTGGTCCAGCACGTCTTG-3' |
| ***WNT7B* FW** | 5'-AAGCTCGGAGCACTGTCATC-3' |
| ***WNT7B* Rev** | 5'-ACTGGTACTGGCACTCGTTG-3' |

**Table S1.** **Primer sequences used for qRT-PCR.** Primer sequences used for qRT-PCR are shown. FW, forward primer; Rev, reversed primer.

| **Primer sequences for ChIP-qPCR** | |
| --- | --- |
| **Name** | **Sequence** |
| ***EGFR* FW** | 5´-TTAGGGCAGCTCCTCTTTGC-3´ |
| ***EGFR* Rev** | 5´-ACATGGGAAAGCGAGGAAGG-3´ |
| ***FOS* FW** | 5´-CCATTCACGGGTCCAGACAT-3´ |
| ***FOS* Rev** | 5´-TACAGACAAGCCAGAGGGGT-3´ |
| ***FOSB* FW** | 5´-GCAGGAGGGAGAGGGTAGTT-3´ |
| ***FOSB* Rev** | 5´-GATGTCAGCGGCCAGGTATG-3´ |
| ***FOSL1* FW** | 5´-AATGATGTAGCAAGCGCCCA-3´ |
| ***FOSL1* Rev** | 5´-TCCCAACCCACTCCTCAGAT-3´ |
| ***HBB* FW** | 5´-AACGTGATCGCCTTTCTC-3´ |
| ***HBB* Rev** | 5´-GAAGCAGAACTCTGCACTTC-3´ |
| ***HBEGF* FW** | 5´-TCCCATTCATGGGGCAACTC-3´ |
| ***HBEGF* Rev** | 5´- CAAACCTAGCTGGGGTGAGG-3´ |
| ***HPRT1* FW** | 5´-TGTTTGGGCTATTTACTAGTTG-3’ |
| ***HPRT1* Rev** | 5’-ATAAAATGACTTAAGCCCAGAG-3’ |
| ***ITGA2* FW** | 5´-GTCCTGCCTCCCAAACACAGGT-3´ |
| ***ITGA2* Rev** | 5´-CCGGTGACACGATTTTGACGCT-3´ |
| ***JUNB* FW** | 5´-AACATCCTGTGGACTGCTGG-3´ |
| ***JUNB* Rev** | 5´-TCACCCTGGGCCATGTTTAC-3´ |
| ***LAMB3* FW** | 5'-TTGCCCTGCACTACAACACA-3' |
| ***LAMB3* Rev** | 5'-GTAACACACCAGGCCCACTT-3' |
| ***MMP2* FW** | 5'-TCCCAGGCCTGCCCATGTCA-3' |
| ***MMP2* Rev** | 5'-GGAGCTGGTGGGTGGAAAGCC-3' |
| ***SMAD7* FW** | 5´-TGGGTTTCGCGGTGGCCATC-3´ |
| ***SMAD7* Rev** | 5´-CGCTCTCCTCCCCTTGCCCT-3´ |
| ***WNT7B* FW** | 5'-TCACCCATGACTCACTTGGC-3' |
| ***WNT7B* Rev** | 5'-AGGTCTCTTCCGCTCTCAGT-3' |

**Table S2.** **Primer sequences used for ChIP-qPCR.** Primer sequences used for ChIP-qPCR are shown. FW, forward primer; Rev, reversed primer.
